# Towards precision home visiting: results at six months postpartum from a randomized pilot implementation trial to assess the feasibility of a precision approach to Family Spirit

**DOI:** 10.1186/s12884-022-05057-4

**Published:** 2022-09-23

**Authors:** Allison Ingalls, Paul Rebman, Lisa Martin, Elizabeth Kushman, Amanda Leonard, Aimee Cisler, Ingrid Gschwind, Amanda Brayak, Ann Marie Amsler, Emily E. Haroz

**Affiliations:** 1 Johns Hopkins University, Bloomberg School of Public Health, International Health Department, Center for American Indian Health, Allison Ingalls, 415 N. Washington St., 4th Floor Room 439, Baltimore, MD 21231 USA; 2 Inter-Tribal Council of Michigan, Inc, Sault Ste. Marie, MI USA; 3 American Indian Health and Family Services, Detroit, MI USA; 4 Tribal Communities in Michigan, MI, USA

**Keywords:** Home visiting, Precision, Precision prevention science, Precision home visiting, Implementation, Hybrid design

## Abstract

**Background:**

Shared implementation challenges at scale in early childhood home visiting have led researchers to explore precision home visiting as a promising service delivery mechanism to better address families’ unique needs and build greater program efficiencies. This randomized controlled pilot study aimed to assess the acceptability of a precision approach to one home visiting model, Family Spirit® and explore potential differences between Precision Family Spirit (PFS) and Standard Family Spirit (Standard FS) on participant-home visitor relationship and maternal outcomes.

**Methods:**

Participants (*N* = 60) were at least 14 years old, pregnant or within 2 months postpartum, and enrolled in Family Spirit. Four sites in Michigan were randomized 1:1 to deliver PFS (up to 17 core lessons plus up to 13 additional lessons as needed) or Standard FS (home visiting services as usual). Primary (program acceptability, participant satisfaction, home visitor-participant relationship quality, retention, adherence) and secondary (knowledge, quality of life, difficulty with parenting problems, substance use, depression, stress) outcomes at 6 months postpartum are presented. PFS participants also self-reported on quality of life, difficulty with parenting problems, stress, substance use, and concerns with sexual and reproductive health and self and child’s nutrition status at each home visit. This informed which lessons they should receive.

**Results:**

Mothers in both groups reported positive program acceptability, satisfaction, and home visitor-participant relationships at 6 months postpartum. However, open-ended feedback from Standard FS participants indicates that some lesson content may not be applicable to all participants. At 6 months, retention was 82.3% for PFS and 66.7% for Standard FS, and adherence was 30.1% for PFS and 20.6% for Standard FS.

**Conclusions:**

Preliminary findings indicate that precision home visiting may be acceptable and feasible. A definitive trial is needed to build on this pilot data, assess outcomes for mothers and children participating in a precision approach to home visiting as compared to standard home visiting, and ready this approach for scale.

**Trial registration:**

ClinicalTrials.govNCT03975530 (first posted on 05/06/2019).

## Background

Early childhood home visiting has supported families’ health and well-being for decades. Since 2010, bipartisan, federally legislated funding for home visiting has been available for states, tribes, and territories through the Maternal, Infant, and Early Childhood Home Visiting (MIECHV) Program. The American Academy of Pediatrics has also documented its support of sustained federal funding for early childhood home visiting to improve maternal and child health and well-being [1].

As the field of early childhood home visiting continues to expand, so does the need for efficient implementation research aimed at understanding what elements of a model are critical to continued effectiveness when delivered in practice. In the most robust analysis of implementation of national evidence-based home visiting models, the Mother and Infant Home Visiting Program Evaluation (MIHOPE) and MIHOPE-Strong Start [2, 3] findings revealed that most programs struggle with low retention rates and a substantial reduction in effects when compared to the original trials. The COVID-19 pandemic has exacerbated implementation challenges. A recent report highlighting the caregiver perspective on home visiting during the pandemic found that home visiting service delivery models must find ways to allow for flexibility during implementation in order to remain effective [4].

Precision home visiting (PHV) is a new field of research aimed at determining what elements of home visiting work best for families in particular contexts. Drawing on principles of precision medicine and precision public health, and through the lens of precision prevention science [5], PHV is aimed at differentiating what works, for whom, and in what contexts to understand how implementation of home visiting services can be tailored to families’ unique needs [6].. The limited body of literature available on PHV has focused on providing encouragement for research in PHV or on informing the design of approaches to PHV with little to no focus on how to administer these strategies in practice [5–7]. Leveraging precision principles to inform home visiting service delivery has emerged as a promising approach to addressing the shared implementation challenges of engagement and retention when bringing evidence-based home visiting interventions to scale [6, 8]. If we can better deliver home visiting services that are matched to the individual families’ circumstances, they will be more likely to engage with and benefit from services. Indeed, a tailoring of services and a collaborative approach to home visiting service delivery has been found to increase retention and the number of completed visits among participants [9, 10].

The Johns Hopkins Center for Indigenous Health (CIH; formerly Center for American Indian Health) has been supporting Native American and some non-Native communities across the United States in implementing its evidence-based home visiting model, Family Spirit®. Scale-up of the model began after the completion of three randomized controlled trials (RCT) conducted to test Family Spirit’s efficacy, through a paraprofessional delivery mechanism, to promote optimal health and well-being for mothers and their child from pregnancy to the child’s third birthday [11–14]. Each RCT corroborated previous results, and data from the largest, most rigorous RCT led to Family Spirit being approved by the Home Visiting Evidence of Effectiveness review in 2014 [15]. Currently, Family Spirit is the only home visiting model categorized as evidence-based for tribal communities that is approved for use with funding from the MIECHV Program.

Informal tailoring of home visiting services, including Family Spirit, has been done for years in Indigenous communities. Except for Family Spirit, home visiting models have not been rigorously tested for effectiveness in these populations, making tailoring to population and context necessary. Even with Family Spirit, affiliates often describe how their best home visitors tailor services to meet their families’ unique needs, making positive outcomes more likely. These discussions sparked new research to develop a precision approach to Family Spirit as an implementation strategy and test it empirically. The goal of such an approach is not to dismiss the informal tailoring that has long existed but to provide all home visitors, even those with less experience, with empirical tools to make consistent changes to program delivery and achieve intended outcomes for the parents and children they serve.

Our team has spent the past 4 years informing a precision approach to home visiting as an implementation strategy to address implementation challenges  [16, 17]. Initial work involved a secondary data analysis of Family Spirit trial data to identify who benefited most from the program [16]. Children of enrolled mothers who had a history of substance misuse benefitted most from the intervention. In addition, the following socioeconomic indicators moderated intervention effects: unstable housing, first-time mothers, and low educational attainment. To complement this secondary data analysis, and in the absence of empirical data, we also engaged Family Spirit stakeholders (home visitors, program managers, and model developers) to identify core components of the Family Spirit model based on their experience [17]. Their input helped define the curriculum pathways that would be used to make the model more precise for families being served. Finally, to facilitate operationalization and implementation of this formative work, we leveraged measurement-based care (MBC) strategies, whereby brief measures of specific outcomes are administered frequently, to help guide home visitor decision making on individual service provision [18, 19]. Broadly, MBC strategies have been shown to significantly improve patient/client level psychosocial outcomes [18, 19], but have never been applied in the home visiting field. Applying MBC to home visiting means systematically measuring pre-specified individual outcomes before each visit, in this case maternal mental and behavioral health outcomes.

This formative work led to an implementation study, the first of its kind in the context of home visiting, which is the focus of this paper. The aims of this trial were to: 1) explore the acceptability and feasibility of a precision approach to home visiting; 2) explore differences in the client-home visitor relationship between both arms; and 3) gather information on family outcomes to inform study design and other features for a fully powered study.

## Methods

### Pilot study design

A multi-site, hybrid type III implementation research design [20] was used to explore, through a pilot study, the utility of a precision approach to delivering the Family Spirit model. Hybrid study designs allow researchers to combine components of both effectiveness and implementation study designs. According to Curran and colleagues’ typology, a hybrid type II implementation research design focuses on testing an implementation strategy while also observing specified outcomes [20]. This paper includes data collected in the first 6–9 months of enrollment in a home visiting program (pregnancy to 6 months postpartum). As previously described [21], the implementation strategy was adapted from evidence-based techniques used in psychotherapy to achieve greater impact [22]. In this study, we applied a similar design concept, called modularity, to pilot a precision approach to home visiting. In short, modularity defines “building blocks” of an intervention that can be combined in different ways to meet unique needs of different individuals within a population [22, 23]. –This implementation strategy was guided by a conceptual model of implementation research developed by Proctor and colleagues, which relies on a taxonomy of eight implementation outcomes [24]. Table 1 in the published protocol for this pilot study provides the specification of the implementation strategy used in this study [21]. Four Family Spirit home visiting sites, from tribal communities/ organizations engaged in the Inter-Tribal Council of Michigan home visiting network, were selected to participate based on comparability (e.g., number of clients served and geographic similarity) and willingness to conduct a pilot study within their existing home visiting programs. Tribal communities and participating organizations are not named in this manuscript out of respect for their wishes to remain anonymous. Two study sites are in the Upper Peninsula and two are in the Lower Peninsula in Michigan. In addition, two study sites are in rural areas, one is urban, and one is suburban. Prior to enrollment, participating study sites were randomly assigned to deliver either Precision Family Spirit (PFS) or Standard Family Spirit (Standard FS). Sites were matched based on comparability (e.g., geographic location, number of clients), and the Principal Investigator flipped a coin to assign each site to deliver either PFS or Standard FS. In their regular home visiting programming, PFS study sites served a combined 33 prenatal clients from May through December 2018, the period before the study began, and Standard FS study sites served 34 prenatal clients during the same period. Prenatal clients served was the indicator used to compare size of home visiting programs at each study site. Postpartum clients were not included during this formative phase. Only PFS home visitors were trained in protocols for delivering the precision approach to Family Spirit. A total of 14 home visitors/supervisors participated in the initial pilot study protocol training in April 2019. One trained PFS home visitor retired before the study launched. While Family Spirit was designed to be delivered by paraprofessional home visitors, many implementing agencies today employ credentialed professionals as home visitors. For this pilot study, nearly all trained home visitors are credentialed professionals. All trained home visitors delivered PFS or Standard FS in their respective sites. Home visitors were not blind to group assignment of study participants, but all study participants were blind to group assignment. Detailed pilot study methods have been published previously [21]. This manuscript reports results from pregnancy or prior to 2 months postpartum and through 6 months postpartum. In reporting the results of this randomized pilot implementation trial, we followed the CONSORT 2010 statement: extension to randomised pilot and feasibility trials [25].

**Table 1:** Standard Family Spirit and Precision Family Spirit lesson schedules, pregnancy to 6 months postpartum

	**Standard Family Spirit (Family Spirit “As Usual”)**	**Precision Family Spirit**				
**Prenatal Period**						
**Timepoint (weeks gestation)**		**Core**	**First-time mother**	**Substance misuse**	**Early childhood obesity**	**Sexual/ reproductive health**
28 weeks	Contributing to a Healthy Pregnancy	Contributing to a Healthy Pregnancy				
29 weeks	Working Towards a Better You			Effects of Drug Use on a Developing Baby		
30 weeks	Changes a Woman Goes Through^a^	Working Towards a Better You				
31 weeks	A Look at Drug Use in Our Community^b^				What You Eat = Your Baby’s Future^c^	
32 weeks	Effects of Drug Use on a Developing Baby	Bedtime Safety				
33 weeks	Understanding Gestational Diabetes^a^					
34 weeks	Baby Proofing and Safety Inside and Outside the Home^b^	How to Feed				
	Bedtime Safety					
35 weeks	How to Feed		How to Diaper			
			How to Dress			
			How to Bathe			
36 weeks	How to Diaper	Breastfeeding Basics				
	How to Dress					
37 weeks	Breastfeeding Basics (Before You Begin / Tips)		Preparing for Safe Travel			
	How to Bathe		Before and During Labor			
38 weeks	Before and During Labor	After Your Baby is Born				
	Time to Push					
39 weeks	Preparing for Safe Travel and Outings					
	Understanding Paternity^a^					
	**Standard Family Spirit (Family Spirit “As Usual”)**	**Precision Family Spirit**				
**Postpartum Period**						
**Timepoint (weeks postpartum)**		**Core**	**First-time mother**	**Substance misuse**	**Early childhood obesity**	**Sexual/ reproductive health**
1 week					Feeding Support^c^	
2 weeks	After Your Baby is Born	How to Comfort Your Crying Child				
	How to Comfort Your Crying Child					
3 weeks	Understanding Reproduction		What to do if Your Baby is Sick			
	How to Protect					
4 weeks	Effects of Drug Use on Our Families and Loved Ones^b^	Parenting Techniques Part A^d^				
5 weeks	Your Family Planning Options					Understanding Reproduction
6 weeks	Planning Ahead	How to Protect				
7 weeks	What to Do if Your Baby is Sick					Your Family Planning Options
	What are Immunizations and Why Do We Need Them?^a^					
8 weeks	More About Immunizations^a^	Planning Ahead				
9 weeks	Parenting Techniques				Rethink that Drink^c^	
10 weeks	Protecting Your Sexual Health^b^	Playtime Fun				
		Protecting Children from Abuse and Neglect				
11 weeks	Learning More about STIs^b^					
12 weeks	Protecting Children from Abuse and Neglect	Parenting Techniques Part B^d^				
	Playtime Fun and Learning					
14 weeks	Introduction to Oral Health Care	Introduction to Oral Health Care				
	Oral Health Care: Getting a Healthy Start^a^					
16 weeks	Introducing Solid Foods to Your Baby	Introducing Solid Foods to Your Baby				
18 weeks	Communication and Building Healthy Relationships	Communication and Building Healthy Relationships				
20 weeks	Skills for Healthy Living Part A	Skills for Healthy Living Part A				
22 weeks	My Health and My Family’s Health^a^				Infant Physical Activity and Safe Play Space^c^	
24 weeks	Skills for Healthy Living Part B	Skills for Healthy Living Part B				

^a^ These lessons are prescribed in Standard Family Spirit but not included in Precision Family Spirit

^b^ These lessons are prescribed in Standard Family Spirit before 6 months postpartum, but they are prescribed to Precision Family Spirit after the six-month postpartum timepoint

^c^ These lessons are prescribed in Precision Family Spirit but not included in Standard Family Spirit. They were developed as part of a new module for the Family Spirit home visiting program

^d^ Parenting Techniques is one lesson that may be taught over two visits. For the purposes of this study and reporting of results, it is counted as one lesson

### Participants

Eligible participants were women who were pregnant or less than 2 months postpartum, had conceived the study baby when they were at least 14 years old, and were already enrolled in Family Spirit home visiting services in their community. Participants were ineligible to participate if they did not meet any of the inclusion criteria previously listed or were unwilling to participate in the full study, including all evaluation components. Participants were screened for eligibility prior to approaching them to enroll in the pilot study, following local recruitment procedures that were in place prior to introducing the study into the existing home visiting program. This typically meant that study teams referred to participant records to identify potentially eligible participants. Then, they attempted contact to review the study and seek consent to participate. For any potential participants who declined to participate in this study, they could continue with regular home visiting services. Study staff gave each potential participant an overview of the study and obtained informed consent from any woman who expressed interest in enrolling after the study was fully described to her. All the enrolled participants ended up being 18 years old or older at enrollment, so assent and parent consent were not necessary for this pilot study, but approved forms were available for home visitors to use if needed. The study was approved by the Johns Hopkins Bloomberg School of Public Health Institutional Review Board (BSPH IRB). Given the low risk of the research and existing comprehensive review and approval by BSPH IRB, appropriate authorities at each participating site provided a support letter affirming their review and approval of the research protocol.

### Standard Family Spirit (Family Spirit “as usual”)

The Family Spirit model includes 63 curriculum lessons that may be delivered to a caregiver from pregnancy until a child’s third birthday. Because this paper presents results from pregnancy until their child was 2 months old and until 6 months postpartum, up to 40 of the 63 Family Spirit lessons were added to Standard FS mothers’ lesson schedules, depending on when enrollment took place (prenatally or postpartum) (Table 1). These lessons were scheduled according to the standard sequence of lessons advised by the Family Spirit model developers. Visits are intended to be scheduled weekly in the prenatal period and through 12 weeks postpartum and bi-weekly from 3 to 6 months postpartum. For any study participants who enrolled in the postpartum period, home visitors decided whether they wanted to schedule additional visits to cover relevant lessons from the prenatal standard sequence of lessons. However, this was not a requirement, and those lessons were not considered part of that participant’s prescribed set of lessons. Seven home visitors, two at one Standard FS study site and four at the other Standard FS study site, were trained in the pilot study protocol for the comparison arm of the trial. Standard FS home visitors were also trained in an implementation support platform called Care4 [26] for data collection, but they did not receive any notifications related to altering the curriculum delivery based on participant data.

### Implementation strategy (precision Family Spirit)

Formative work involved surveying Family Spirit implementers and a secondary data analysis of prior Family Spirit research data to determine a set of core lessons and additional lesson pathways used in this pilot trial [16, 17]. It is important to note that some Standard FS lessons prescribed in the prenatal through 6 months postpartum period were excluded from the precision approaches lesson schedule. This was based on feedback gathered as part of the formative process to design PFS. PFS participants were prescribed a core set of Family Spirit lessons (up to 17, depending on whether the mother enrolled prenatally or postpartum before the baby was 2 months old) (Table 1). For example, if a mother enrolled in the study when her baby was 4 weeks old, the home visitor would not be required to teach any of the lessons from the prenatal period but would need to fit in the one core lesson from the postpartum period that she missed and up to two additional lessons (one from the first-time mother lesson pathway and one from the early childhood obesity lesson pathway). Depending on mothers’ needs identified through assessment, up to four additional sets of lessons were added to the participants’ course plans. For the purposes of this pilot study and analysis, all lesson pathways outlined in Table 1 are developmentally appropriate from pregnancy through 6 months postpartum. Through prescribed curriculum lessons, these additional lesson packages addressed topics from pregnancy through 6 months postpartum related to four areas of self-reported need or concern: 1) being a first-time mother (six additional lessons), 2) identified substance misuse (one additional lesson), 3) nutrition concerns for themselves or their baby (four additional lessons), and 4) sexual/ reproductive health concerns (two additional lessons). Mothers could be assigned to more than one additional lesson pathway. Depending on which lesson pathways a mother was assigned to, they could receive up to 13 additional lessons for a potential total of up to 30 lessons by 6 months postpartum.

Seven home visitors, four at one PFS study site and three at the other PFS study site, were trained in the precision approach to delivering Family Spirit. Home visitors were also trained in Care4 and received alerts through Care4 when participants needed a certain sequence of lessons to be added to their course plan. Alerts were triggered by data collected at baseline and each of the major assessment timepoints (2 and 6 months postpartum), as well as at each home visit when a session summary form was completed. Along with collecting routine visit data and consistent with an MBC approach, each session summary form included a 5-minute self-report questionnaire completed by the participant each time she had a lesson with her home visitor. Standard FS home visitors also collected routine visit data from their participants, but they did not collect participant-reported outcome data since the study team was mainly interested in using this information for systematic tailoring of content. Responses to certain questions on the main assessment questionnaires and the session summary forms triggered the alerts to the home visitors through an email and “insight” (alert on the participant’s case page in Care4) in the implementation support platform. For example, if a study participant provides information that puts them at high risk for substance misuse, the home visitor received the following message “{Client #} indicated a high risk of substance abuse. Apply substance abuse [lesson pathway] to this case.” Table 2 provides a complete overview of what assessment questions triggered lesson pathways for PFS study participants. Prescribed PFS lessons were taught in-person or by phone, when COVID-19 pandemic restrictions were in place. Each lesson typically takes between 30 and 60 minutes to complete. Additionally, in both groups, home visitors were notified by Care4 when their participants posed a risk of self-harm, screened positively for depression, or reported concerns about housing/ homelessness, so that appropriate referrals and support could be provided.

**Table 2:** Precision Family Spirit implementation strategy participant assessment timepoints and variables to determine lesson pathways

Assigned lesson pathway	Trigger for lesson pathways	Baseline	2-month assessment	6-month assessment	Session summary form^**a**^
First-time mother	Positive response (YES) to “Are you a first-time mom?”	x			
Substance misuse (high risk)	Screen positive on the modified ASSIST for alcohol or other drug misuse Screen positive on the ASSIST is: Alcohol score of 11 or above Illegal drugs score of 4 or above Legal drugs (not as prescribed) score of 4 or above Injection drug use 1 or above	x	x	x	
	Positive response (YES) on the IHR 5Ps “Do any of your friends have a problem with alcohol or other drug use?” OR Positive response (YES) to “Does your partner or significant other have a problem with alcohol or other drug use?” AND Positive response (YES) to “Since our last visit, have you drunk any alcohol or used other drugs?” AND 1 or more times in the last month they had 4 or more drinks	x	x	x	x
Substance misuse (medium risk)	Positive response (YES) on the IHR 5Ps “Do any of your friends have a problem with alcohol or other drug use?” OR Positive response (YES) to “Does your partner or significant other have a problem with alcohol or other drug use?” AND Positive response (YES) to “Since our last visit, have you drunk any alcohol or used other drugs?”	x	x	x	x
Early childhood obesity	Positive response (YES) to “Do you have concerns about your nutrition?”	x			
	Positive response (YES) to “Since the last time you had a visit for this program, have you worried about what and/or how much you and/or your child is eating?”		x	x	x
	Home visitor positive response (YES) to “Are you worried about what and/or how much this participant and/or her child is eating?”				x
Sexual/reproductive health	Positive response (YES) to “Since the last time you had a visit for this program, have you had concerns about your own sexual or reproductive health?”		x	x	x
	Home visitor positive response (YES) to “Do you have any concerns about this participant’s sexual or reproductive health?”				x

^a^ The session summary form is an in-visit form used to collect routine visit data for all study participants. For Precision Family Spirit participants only, they also completed self-report on these specified outcomes to inform systematic tailoring of the curriculum content

### Data collection and primary outcomes

Quantitative assessments were administered in participants’ homes, by phone, in other private locations, or via SMS/email link sent to the participant. Data was collected either on paper or electronically via Care4 (hosted on a HIPAA-compliant server) on any of the following devices: tablet, laptop computer, or smartphone (in the case of assessment links sent via SMS to participants). Data collection included self- and parent-report questionnaires that participants self-administered if in-person or via SMS/email link and home visitors administered as interviews if over the phone. Any self-administered data collection was automatically recorded into Care4 so that the home visitors did not see participant responses. For visits done over the phone, the home visitor recorded participant responses into the data system. Data was collected by the same home visitors who delivered lessons to study participants, except for self-report data collected on the home visitor-participant relationship domain. Those data were collected separately by phone with a supervisor. Secondary data analysis of existing programmatic data was used to complement primary data collection for the analysis of program retention and adherence. Enrolled mothers were given a $15 gift card at the completion of the baseline self-report assessment. They received a $15 gift card at the 2-month and 6-month assessments.

At each visit, participants were asked whether they or their baby visited the Emergency Department or were hospitalized since the last visit. Home visiting staff took detailed notes, and the Project Manager worked with a Regulatory Specialist to report all adverse events to the BSPH IRB. None of the reported adverse events were found to be related to participants’ involvement in the pilot study.

#### Primary outcomes measures

This was a pilot study aimed at evaluating the feasibility of an implementation strategy designed as a precision approach to Family Spirit implementation. In addition to examining the feasibility of recruitment (number approached and number enrolled per month), randomization (proportion of those approached and eligible who enroll), and assessment procedures (proportion of planned assessments completed) [27], the following primary outcomes were defined: program acceptability, participant satisfaction, home visitor-participant relationship, and home visiting services retention and adherence at 6 months. Self-reported program acceptability was measured at 6 months postpartum with a 15-item instrument developed by researchers at Johns Hopkins University to measure implementation of mental health programs in low resource settings. Items included in this instrument are based on leading implementation science frameworks (Consolidated Framework for Implementation [CFIR]; Reach, Effectiveness, Adoption, Implementation, and Maintenance [RE-AIM]; and a conceptual model of evidence-based implementation in public services sectors [EPIS]) [24, 28–30], as well as knowledge gathered from health systems, dissemination, and implementation science experts. The instrument has demonstrated good psychometric properties across a range of stakeholder groups in low-income settings internationally (alpha = 0.79 and alpha = 0.77) [31]. Each item is scored on a 4-point ordinal scale ranging from 0 “not at all” to 3 “a lot,” with an additional category for “don’t know/not applicable” with higher total scores indicating more acceptability. Items cover participant perspectives on program acceptability as it relates to the model itself (i.e., Family Spirit) and home visiting services generally provided to them. Sample questions from this instrument include, “Do you feel like the lessons of Family Spirit make sense to you?”, “Do you feel that your home visitor is qualified to deliver Family Spirit?”, and “Do you feel that you understand the way things are explained to you during Family Spirit?”

Program satisfaction was measured at 6 months postpartum using a 34-item instrument adapted by the study team from a similar measure used during the most recent Family Spirit efficacy trial [12]. For the purposes of this study, we focused on the seven items that aimed to measure satisfaction with the skills and competencies Family Spirit aims to instill in parents (e.g., “I learned helpful skills” or “Because of what I’ve learned in the program, I feel that my child is healthier”). The satisfaction items were scored 0 “Strongly Disagree” to 5 “Strongly Agree,” and higher total scores correlated with higher levels of satisfaction. In addition, this study also focused on open-ended responses to the following items related to program feedback: 1) “What was the most helpful part of the program? Please explain why.” 2) “What was the least helpful part of the program? Please explain why.”

Home visitor-participant relationship was measured using a short version of the Working Alliance Inventory (WAI), a valid and reliable tool for measuring therapeutic alliance [32–34]. The WAI has been used in home visiting and measures three domains of the home visitor-participant alliance: 1) agreement between participant and home visitor on the goals of the treatment (Goal; e.g., My home visitor and I are working toward mutually agreed upon goals.); 2) agreement between participant and home visitor about the tasks to achieve these goals (Task; e.g., What I am doing with home visiting gives me new ways of looking at my family’s situation.); and 3) the quality of the bond between the participant and home visitor (Bond; e.g., feel that my home visitor appreciates me.) [35, 36]. Possible scores for each item on the WAI ranged from 1 “Never” to 7 “Always” and are summed for a total score. A higher score on the WAI indicates a stronger therapeutic alliance.

Consistent with other studies in the field, home visiting services retention was defined as the percent of participants who were still enrolled in home visiting services at 6 months after enrollment into the study [3]. Participants were considered enrolled if they completed a visit in the subsequent month. Participants who did not complete at least one home visit were excluded from the retention analysis. Participants were still enrolled in the research study regardless of their retention in home visiting services. Dosage was calculated as the number of visits completed from study enrollment to 6 months post enrollment for all participants. Finally, home visiting services adherence was defined as the percent of prescribed lesson visits from birth to 6 months postpartum that were completed.

#### Secondary outcomes measures

Secondary outcomes measured across both groups at baseline, 2, and 6 months postpartum included parent knowledge, substance use, maternal depression, and quality of life. Parenting stress and difficulty with top parenting problems (a participant defined measure) was only measured among PFS participants at baseline, 2, and 6 moths, and at the beginning of each session as part of their session summary forms. Parent knowledge was measured at baseline, 2, and 6 months postpartum through a short (10 multiple choice and true/false items) knowledge assessment developed by the study team to assess maternal knowledge through a series of curriculum-based knowledge questions (e.g., “Which of the following is the best way for a parent to encourage early learning?” and “Which of these is NOT a sign of dehydration in babies and young children?”).

Maternal substance use was measured using an adapted version of the World Health Organization’s Alcohol, Smoking, and Substance Involvement Screening Test [37]. This questionnaire covers 10 main substance groups and screens for all levels of problem or risky substance use (alcohol, illegal drugs, and prescription drugs). A risk score is provided for each substance, and scores are grouped into low, moderate, or high risk. The Edinburgh Postnatal Depression Scale was used to measure maternal depression. It is a 10-item self-rating scale that was specifically designed for women who are pregnant or have just had a baby; however, it has also been shown to be an effective measure for general depression in the larger population [38, 39].

Quality of life was measured using the visual analog scale from the EQ-5D to measure participants’ self-reported quality of life. It records the respondent’s self-rated quality of life on a vertical visual analog scale (labeled 100 indicating “The best quality of life you can imagine” to down to 0 indicating “The worst quality of life you can imagine”). For parenting challenges, we used an adapted version of the Top Problems scale [40]. Participants were asked at baseline about the biggest challenges they are worried about right now related to parenting. Up to 10 challenges were listed and then the participant was asked to go back and rate how big a challenge each of the problems was for them using a scale of 0 “Not at all challenging” to 10 “Very challenging.” At 2 and 6 months postpartum, the participant then rated how much difficulty they had with the three most challenging problems using a scale of 0 “No difficulty” to 3 “A lot of difficulty” since their last visit. Additional challenges were also solicited at each assessment time point. This measure allowed for measuring progress on client-defined parenting goals. Finally, parental stress was measured using the short (4 items) version of the Perceived Stress Scale [41]. Items range from 0 “Never” to 4 “Very Often” with higher total scores correlated to more perceived stress.

### Power and sample size

As this was a pilot and feasibility study, the sample size was based on pragmatics of recruitment and what was necessary to examine feasibility. We did explore hypothesis testing between groups (i.e., PFS vs. Standard FS) as post-hoc analyses, but did not base sample size calculations on use of inferential statistical methods. There were no interim analyses or stopping rules.

### Data analysis

Baseline socio-demographic and relevant maternal health characteristics and priorities were examined at baseline across groups. Outcomes were measured using mixed-effects models to account for the nested structure of the data (e.g., observations within people) with the inclusion of the site variable as a random-effect. Models included an interaction term specifying group assignment (0 = Standard FS; 1 = PFS) and the 6-months postpartum time point. We did not impute missing data, as longitudinal mixed-effects models are robust to missing data and multiple imputation may result in unstable results [42]. The proportion of participants retained at 6 months was analyzed using a chi-square test of homogeneity. Adherence was analyzed using an independent-samples t-test. Data cleaning and analysis was done in R [43] and Stata 15 [44].

## Results

**Fig. 1:**
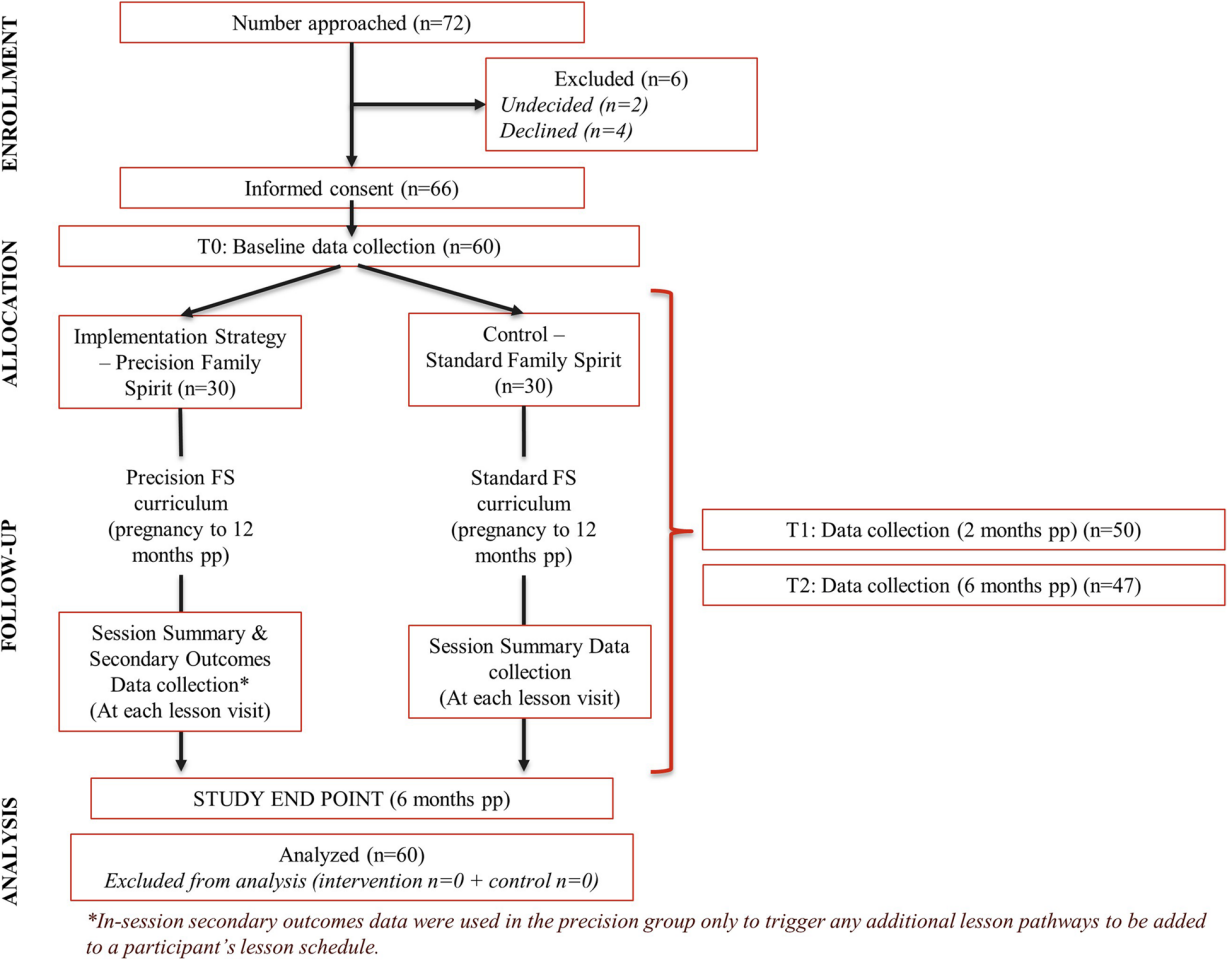
CONSORT trial flow diagram for Precision Family Spirit study. CONSORT flow diagram showing participant flow through each stage of the randomized controlled trial (enrollment, intervention allocation, follow-up, and data analysis). Those who were undecided when the study enrollment period closed were excluded. No participants who completed a baseline were excluded from analysis unless data was missing at a major assessment time point

Of 72 women approached for participation in the pilot study across 5 full months of recruitment, 4 declined to participate, 2 were undecided when study enrollment ended, and 66 consented to participate. Thus, 91.6% of those approached to participate in the pilot study completed informed consent. Of these, 60 completed a baseline assessment between the end of June and mid-December 2019, 83.3% of those who were initially approached and 90.9% of those who gave informed consent (Fig. 1). Follow-up continued through December 2020. Six mothers (PFS*, N* = 3; Standard FS*,* *N* = 3) withdrew during the study period. Fifty participants (83.3%) across both groups completed a 2-month assessment and forty-seven participants (78%) across both groups completed a 6-month assessment.

### Baseline Characteristics

At baseline, participants were on average 26.6 years old (range 18.7 to 38.2 years old), and *n =* 46 were pregnant, while *n =* 14 had a newborn baby less than 2 months old (Table 3). At baseline, most mothers identified as American Indian and Alaska Native (68% [41 of 60]; includes multi-racial mothers). More than half of mothers participating in PFS reported being first-time mothers (53% [16 of 30]), compared to only 9 (30%) of mothers in Standard FS reporting being first-time mothers at baseline.

**Table 3:** Baseline characteristics of participating mothers by group assignment (Precision Family Spirit or Standard Family Spirit)

	PFS (*N =* 30)	Standard FS (*N =* 30)
	N (%)	N (%)
Race ^a^		
AI/AN	20 (67)	21 (70)
Black or AA	7 (23)	0 (0)
White or Caucasian	8 (27)	15 (50)
Don’t know	0 (0)	1 (3)
Identifies as Hispanic	4 (13)	3 (10)
Income category		
50% and under	12 (41)	11 (37)
51–100%	13 (45)	7 (23)
Greater than 100%	4 (14)	12 (40)
Pregnant at Baseline	25 (83)	21 (70)
First-time mother	9 (30)	16 (53)
Ever used alcohol	27 (90)	29 (97)
Ever used drugs	15 (50)	16 (53)
Nutrition concerns	5 (17)	3 (10)
Housing concerns	7 (23)	5 (17)
Age (years), m (*SD*)	26.6 (*5.3*)	26.5 (*5.2*)

Note. *PFS* Precision Family Spirit, *Standard FS* Standard Family Spirit

^a^ Categories for race are not mutually exclusive

### Primary outcomes

#### Program acceptability

Overall, clients in both PFS and Standard FS rated the program as highly acceptable; there were no statistically significant differences between the groups (Table 4).

**Table 4:** Primary outcomes of the Precision Family Spirit pilot implementation trial, 6 months postpartum (*N* = 60)

	Standard Family Spirit	Precision Family Spirit	
	Average score at 6 months (SE)	Average score at 6 months (SE)	Between group difference at 6 months [95% CI]
Acceptability	2.97 (0.02)	2.89 (0.06)	0.07 (0.06) [−0.05, 0.20]
Satisfaction	29.2 (0.53)	29.0 (0.74)	0.22 (0.90) [−1.60, 2.04]
Home visitor relationship	77.8 (1.39)	77.3 (1.29)	0.48 (1.91) [−3.37, 4.32]
	% (N) [95% CI]	% (N) [95% CI]	*p*-value^a^
Home visiting services retention (*n* = 59)	82.3% (24) [69.0, 96.5%]	66.7% (20) [49.8, 83.5%]	0.26
Participant adherence	20.6% (17.1%) [14.5, 26.7%]	30.1% (24.9%) [21.2, 39.0%]	0.09

^a^ Authors acknowledge that the study is not adequately powered as this is a pilot study [45]

#### Participant satisfaction

Clients in both groups also reported higher than average levels of satisfaction with the program. There were no statistically significant differences between groups at 6 months postpartum. Participants in both groups shared open-ended feedback about specific curriculum content that was most helpful. Responses centered on child development, breastfeeding and child feeding, and routines/monitoring. For example, one Standard FS participant said, “The most helpful part is learning and understanding what my child needs from me right now.” A PFS participant shared, “Information to prepare me for breastfeeding and parenting...have allowed me to be more confident in my choices.” Referrals to resources in the community was also a shared component of participant satisfaction. One PFS participant appreciated “referrals to get the right help where needed.” A Standard FS participant liked “the referrals to other programs and the advice given.”

Participants were also asked about what was least helpful in the program or challenges they had in participating. Standard FS participants shared some insight into what standard curriculum content felt unnecessary, including “lesson plans that contain knowledge I already have;” “repetition of information;” and “required lessons are things I already know.” Another Standard FS participant said, “Some lessons have been what seems to me is “common sense,” and I feel as if those aren’t very helpful to me. But I understand not all moms-to-be are as informed as I am.” Finally, one of the participants provided a specific example of content that did not feel particularly useful by responding, “Drug information- Hasn’t been helpful because I don’t do any of these things, so they do not pertain to me.” There were no similar comments from the PFS participant responses to this same question about program content.

#### Home visitor-participant relationship

The relationship between the home visitor and participant were also rated highly across both groups, indicating strong therapeutic alliances. There was no statistical significance between groups.

#### Home visiting services retention

A total of 30 (100.0%) Standard FS participants and 29 (96.7%) PFS participants completed at least one visit. Of those that completed at least one visit, retention in home visiting services at 6 months was 66.7% (*N* = 20) for PFS participants and for 82.3% (*N* = 24) Standard FS participants. Retention rates did not differ by group at 6 months (*p* = 0.26). Among participants who reached 6 months in the program before COVID-19 restrictions were put in place, retention was 70.0% (*N* = 14) for PFS participants and 85.0% (*N* = 17) for Standard FS participants. For dosage, PFS participants received an average of 7.0 (SD = 3.1; *Range:* 1–14) visits and Standard FS participants received an average of 8.2 (SD = 4.3; *Range:* 0–20) visits during their first 6 months in the program.

#### Adherence to lessons as prescribed

Before 6 months postpartum, all 30 PFS participants were assigned the core lessons, 9 (30.0%) were assigned to the first-time mother lessons, 10 (33.3%) were assigned the nutrition lessons, 8 (26.7%) were assigned the substance abuse lessons, and 6 (23.3%) were assigned the sexual/reproductive health lessons. These additional assignments were not mutually exclusive, meaning any participant could be assigned to more than one additional lesson pathway. A total of 17 (56.6%) of participants were assigned to at least one additional lesson pathway, and the average number of additional lesson pathways assigned to PFS participants from enrollment until 6 months postpartum was 1.13 (SD = 1.20).

Between birth and 6 months postpartum, PFS participants were assigned an average of 12.3 (SD = 1.80; *Range:* 11–16) lessons. All Standard FS participants were assigned 22 lessons from birth to 6 months postpartum. PFS participants completed an average of 3.63 assigned lessons or 30.1% (SD = 24.9%) of their assigned lessons. Standard FS participants completed an average of 4.53 assigned lessons or 20.6% (SD =17.1%) of their assigned lessons. There was no statistically significant difference between the two groups in adherence at 6 months (*p* = 0.09). A total of 8 Standard FS participants and 9 PFS participants completed their six-month assessments prior to COVID-19 restrictions prevented in-person visits from occurring. For Standard FS who completed their six-month assessment before COVID-19 restrictions, the mean percentage of lessons completed was 33.0%, compared to 16.1% among Standard FS participants who completed their six-month assessment after COVID-19 restrictions were implemented. A similar pattern was true for PFS participants. PFS participants who completed their six-month assessment before COVID-19 restrictions were in place completed an average of 33.1% percent of lessons compared to 28.8% for participants who completed their 6 months assessment after COVID-19 restrictions were implemented.

### Secondary outcomes (Table 5)

There were no statistically significant differences between groups on change in depression symptoms, alcohol or substance use, or parenting knowledge (Table 5). Among PFS participants only, using data from the participant monitoring forms, we observed that difficulty with participant defined parenting challenges decreased significantly from baseline through 6 months (*p <* 0.001). No significant decrease was observed for parenting stress over the course of participation in PFS.

**Table 5:** Secondary outcomes of the Precision Family Spirit pilot implementation trial, 6 months postpartum

	Standard Family Spirit	Precision Family Spirit	
	Difference in score from baseline	Difference score from baseline	Between group difference in mean change from baseline [95% CI]
Maternal depression	0.95	−0.30	−1.24 (1.42) [−4.03, 1.54]
Parental alcohol use	1.1	0.29	−0.81 (0.81) [−2.38, 0.77]
Parental substance use	−2.16	−2.23	−0.08 (1.39) [−2.81, 2.65]
Parenting knowledge	0.14	0.35	0.21 (0.42) [−0.63, 1.04]
Average change measured over all lessons attended	*P value*^*a*^		
Top parenting challenges	N/A	−0.08 (0.02)	< 0.001
Parenting stress	N/A	−0.02 (0.07)	0.790

^a^ Authors acknowledge that the study is not adequately powered as this is a pilot study [45]

## Discussion

Thanks in part to the MIHOPE study, a legislatively mandated evaluation of the MIECHV program, we know that early childhood home visiting at scale shares implementation challenges [2]. PHV has made its way onto the national research agenda as a promising strategy to address these challenges [5]. This study is the first attempt to test precision home visiting as an implementation strategy and makes several important contributions to the field.

Study participants in both groups were generally satisfied with the home visiting services they received, and acceptability of the program was high as well. Of note, however, is the suggestion by Standard FS participants that the amount of information conveyed to PFS participants was more appropriate compared with the standard prescribed lessons in the Standard FS group. The open-ended feedback from some participants in Standard FS indicated that they felt like some of the prescribed lessons were unnecessary. Further, participants in both groups demonstrated strong relationships with their home visitors indicating that the precision approach did not change the nature of this critical relationship. Taken together, this evidence points to both the promise of precision home visiting, and a need for further testing of the strategy in a fully powered trial, with a possible non-inferiority design.

Participant retention by 6 months was 66.7% for the PFS group and 82.3% for the Standard FS, although differences were not statistically significant. These rates are consistent with or higher than previously found reported national rates, depending on how retention was operationalized (i.e., 39.5–82.3% by 6 months; 46% by 12 months) [3, 46]. They were also higher than rates found by an evaluation of programs in Florida which showed retention of 62.9% 6 months after enrollment [47]. It is important to note, however, that all previous studies on retention rates happened before the COVID-19 pandemic. While not significant, the lower percent retention for PFS participants warrants further consideration. Perhaps the definition that has been previously used in the field of home visiting (considered enrolled if they completed a visit in the subsequent month) and used in this study needs to be revisited as precision approaches to home visiting continue. Because it is expected that home visits will decrease in number, this definition may no longer be capturing retention accurately. Similarly, while not statistically significantly different, adherence rates by 6 months were low for both study groups (30.1 and 20.6% for the PFS and Standard FS groups respectively). Perhaps this is due to the way the four study sites schedule their home visits (e.g., more spread out than the model developer recommends). Variances like this across home visiting implementing agencies must be accounted for in future studies. These rates were also affected by the COVID-19 pandemic, as fewer visits were completed once pandemic restrictions were put in place. These rates may also reflect the differences in volume of content that was prescribed in each approach (e.g., up to 40 lessons for Standard FS and up to 30 lessons but with variation for PFS). The fact that similar outcomes were observed between both groups, despite potentially different dosages and fidelity, warrants further exploration.

On March 11, 2020, the World Health Organization declared COVID-19 a global pandemic, about 8 months after this pilot study launched. The pandemic required nearly all home visiting programs to pivot quickly from in-person visits to exclusively virtual [48]. Participating sites in this pilot study were no exception, and data collection activities remained virtual through the study endpoint. Certainly, sample sizes were too small to detect any meaningful differences between participants who completed their 6-month postpartum assessments before and after the COVID-19 pandemic was declared. We do not fully understand the impact that COVID-19 has had on the field of home visiting broadly and this pilot study specifically.

The question remains of whether PHV is at least equally as beneficial for parents and children as standard home visiting, which can be resource intensive and face implementation challenges related to duration of participation and frequency of home visits. Together the findings from this pilot study can inform a larger fully powered hybrid-implementation study testing the effectiveness of a precision strategy on implementation outcomes while simultaneously monitoring maternal and child outcomes. Given that Family Spirit and similar evidence-based home visiting models are the standard of care, such a trial may be best designed as a non-inferiority trial whereby the hypothesis would be that a precision approach may achieve similar outcomes, but for fewer resources. Indeed, this type of design could work well in studying PHV because of its novelty to the field, well-established evidence of effectiveness of the standard of care (in this case, home visiting models), and call for more research into PHV as a way to address shared implementation challenges in dissemination [49]. It would be important to understand differences more clearly between groups in participant retention and adherence, as well as the costs associated with each type of delivery model. Ultimately, if evidence supports precision approaches to home visiting produce similar outcomes to standard models of care, efficiencies in resource utilization and cost could lead to capacity to enroll more families in need of home visiting services. Consistent with the role and interpretation of pilot studies in clinical research [27], our findings suggest the feasibility of recruitment and randomization, and demonstrate the utility of assessment procedures and modalities for a full powered trial design. This pilot study represents a first step in exploring the application of precision science to home visiting and will inform a larger trial to test the impact of this implementation strategy on implementation of home visiting.

### Limitations

During half of the duration of this pilot study, participating sites were engaged in delivering home visiting services to families amidst restrictions in place due to the COVID-19 pandemic. While “home” visits did continue, most of them occurred virtually (i.e., by phone or video). There has been some preliminary work done on how families’ experiences with home visiting were impacted during the pandemic [49], but there is still a need for further research in this area. Data collected over the phone during the pandemic is also a limitation of this pilot trial because home visitors asked questions interview-style and entered the data themselves. Because this was a pilot implementation trial, we were not able to draw conclusions about maternal outcomes. Our sample sizes were prohibitive of meaningful hypothesis testing. In addition, feasibility results do not necessarily extend beyond the participant profile included in our sample (i.e., inclusion and exclusion criteria which determined participant type). Response bias may exist in participant self-report on the Working Alliance Inventory. For example, participants may report stronger therapeutic alliance to their home visitor if they think the home visitor may see their responses or may always rate their home visitor highly because that is what they think they should report. Home visitors were trained to reduce response bias as much as possible (e.g., having participants complete this measure with a supervisor at a separate time), but we cannot guarantee that bias is nonexistent given the type of measure. The precision approach to home visiting tested as part of this pilot study relied only on assessments. For participants that did not complete a measure that triggered the alerts for specific lessons, there were missed opportunities for prescribing new lessons to a mother’s course plan. In addition, while home visitors were not prohibited from using their own judgment to add new lesson pathways external to the MBC approach, this was not a specific component of the protocol or training. Indeed, home visitors implementing Standard FS could have provided informally tailored content to their participants thus introducing contamination. The research team did not institute formal ways of preventing this in the Standard FS study sites. Future work should consider how to add home visitor judgement as an outcome measure to better understand the interplay between data-driven precision and intrinsic home visitor competencies that inform tailoring of an intervention.

## Conclusions

The COVID-19 pandemic may have impacted the results of this study in ways that we do not understand. Nonetheless, main findings revealed that a precision approach to home visiting may be feasible and acceptable and feedback from Standard FS participants indicate that more precision around content delivery might be useful. We do not yet understand how PHV might impact home visiting services retention and engagement, and more research is needed to learn more. Ultimately, PHV holds promise as a new paradigm to explore.

## Data Availability

The datasets generated and/or analyzed during the current study are not publicly available due tribal data sovereignty over the data but may be available from the corresponding author on reasonable request and with appropriate tribal approvals.

## Abbreviations

BSPH IRB: Johns Hopkins Bloomberg School of Public Health Institutional Review Board
CIH: Johns Hopkins Center for Indigenous Health
MBC: Measurement-based care
MIECHV: Maternal, Infant, and Early Childhood Home Visiting Program
MIHOPE: Mother and Infant Home Visiting Program Evaluation
PHV: Precision home visiting
PFS: Precision Family Spirit
RCT: Randomized controlled trial
Standard FS: Standard Family Spirit
WAI: Working Alliance Inventory
